# Discovering the Forgotten Trauma Behind Chronic Shoulder Pain: The Critical Role of a Thorough Medical Interview

**DOI:** 10.7759/cureus.49173

**Published:** 2023-11-21

**Authors:** Akihisa Watanabe, Takahiro Machida, Katsura Takashima, Takahiko Hirooka

**Affiliations:** 1 Rehabilitation, Machida Orthopaedics, Kochi, JPN; 2 Orthopaedics, Machida Orthopaedics, Kochi, JPN; 3 Orthopaedic Surgery, Onomichi Municipal Hospital, Onomichi, JPN

**Keywords:** acromioclavicular joint/injuries, upper extremity trauma, arthroscopy, chronic pain management, glenohumeral osteoarthritis, shoulder joint pain

## Abstract

We report a rare case of a patient experiencing pain and dysfunction attributable to bone fragments from a trauma sustained over two decades prior. A 43-year-old Japanese woman presented with persistent left shoulder pain. Initial radiographs revealed glenohumeral joint osteoarthritis, an unusual finding for her age. Her medical history included a previously overlooked traumatic dislocation of the left acromioclavicular joint over 20 years ago. Computed tomography scans later uncovered bone fragments below the coracoid process without signs of scapular or tuberosity fractures. The fragments were arthroscopically removed, resulting in significant pain relief. The patient's Shoulder Pain and Disability Index score improved from 60 to 9 at the six-month postoperative follow-up. This case underscores the importance of considering historical trauma in patients presenting with atypical shoulder pain and highlights the potential diagnostic value of revisiting a patient's medical history when unusual lesions are discovered.

## Introduction

While numerous studies have explored various shoulder disorders, such as osteoarthritis (OA), rotator cuff tears, and other pathologies [1], reports linking shoulder pain to bone fragments from past trauma are sparse, with the literature mainly focusing on scapular [2] and lesser tuberosity fractures [3]. In this case, we illustrate how discovering atypical lesions prompted a review of the patient's medical history, revealing a long-forgotten injury as the source of her current pain and dysfunction.

## Case presentation

**Figure 1:**
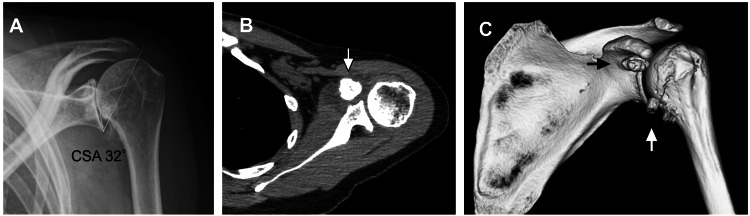
Initial radiograph and computed tomography scans of the patient's left shoulder (A) Radiograph shows the glenohumeral joint osteoarthritis and a critical shoulder angle of 32 degrees; (B) Computed tomography scans show bone fragments (arrow); (C) Three-dimensional computed tomography scans show bone fragments (arrow).

A 43-year-old Japanese woman (height 1.58 m, weight 60.0 kg, body mass index 24.0 kg/m^2^) consulted us for left shoulder pain that had worsened progressively over the preceding two months, affecting her profession as a cook. She reported no recent trauma or other precipitating incidents. Radiographic examination showed glenohumeral OA, which was peculiar given her young age and a critical shoulder angle of 32 degrees (Figure 1A). Magnetic resonance imaging showed no rotator cuff pathology. The unexpected presence of OA prompted a thorough review of her medical history, revealing an almost forgotten traumatic dislocation of her left acromioclavicular joint from over 20 years ago. Computed tomography scans later identified bone fragments under the coracoid process (Figure 1B-1C) with no associated scapular or tuberosity fractures.

**Figure 2:**
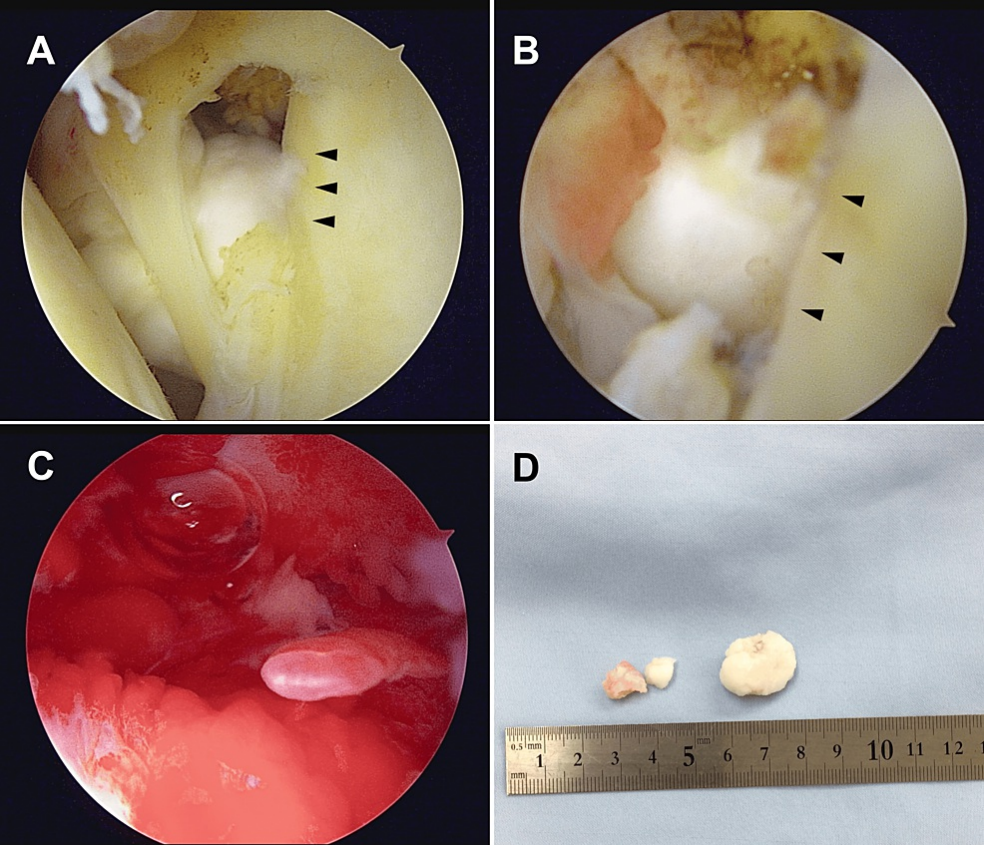
Arthroscopic view and resected bone fragments (A, B) Arthroscopy revealing a large bone fragment embedded inferiorly in the coracoid process (arrowhead); (C) Arthroscopy showing redness around the bone fragment; (D) Resected bone fragments.

During arthroscopic surgery, a large bone fragment under the coracoid process was discovered and excised (Figure 2A-2B). Site redness was noted post excision, which appeared to be the pain source (Figure 2C). Additional smaller fragments were also removed (Figure 2D). Pathologic examination confirmed the fragments as osteophytes due to OA, with no malignant transformation detected. Arthroscopy confirmed that the rotator cuff was intact.

The patient underwent standard postoperative rehabilitation, including range-of-motion and strengthening exercises, supported by celecoxib (100 mg as needed) for pain management. The patient's Shoulder Pain and Disability Index score improved dramatically from 60 preoperatively to nine at the six-month follow-up. She resumed her cooking career three months post-surgery and was able to work with a maximum pain level below three on the 11-point Numerical Rating Scale (Table 1).

**Table 1:** Course of the patient’s function and pain NRS, 11-pont numerical rating scale; SPADI, Shoulder Pain and Disability Index. ^a^ Pain intensity in daily activity at its worst point; ^b^ Average pain intensity in daily activity.

Function/Pain Assessments	Preoperative	Months Postoperative			
		1	2	3	6
Anterior elevation	100	120	155	160	160
Lateral elevation	90	120	145	145	145
Pain at worst (NRS)^a^	6	3	4	3	3
Pain at average (NRS)^b^	6	3	3	1	1
SPADI Score	60	19	22	16	9

## Discussion

The critical observation from this case is the role of bone fragments, likely originating from past trauma, in causing pain and dysfunction. This underscores the importance of a comprehensive review of a patient's historical injuries when faced with unusual shoulder presentations.

In our practice, glenohumeral joint OA is infrequent in the Japanese population [4-6]. This rarity may be partially explained by the fact that individuals from East Asian populations, such as Japan, typically have larger critical shoulder angles than Western populations [7]. A larger critical shoulder angle has been associated with decreased mechanical stress on the glenohumeral joint [8]. In the present case, the patient's critical shoulder angle was 32 degrees, positioning her at a lower risk for developing glenohumeral joint OA, as angles below 30 degrees are considered high risk [9]. Additionally, risk factors for glenohumeral joint OA commonly include aging, Caucasian ethnicity, obesity, and previous trauma [10]. Although rotator cuff arthropathy might have been suspected in the presence of a rotator cuff lesion, no rotator cuff lesion was found in this patient. The discovery of glenohumeral joint OA in this Japanese patient led us to a meticulous reassessment of her medical history, which revealed a significant but nearly forgotten traumatic event.

The significance of investigating prior trauma lies in its potential for causing lasting complications. Although literature regarding post-dislocation bone fragments in the acromioclavicular joint is sparse [11,12], studies on other joints, such as the ankle, have shown that trauma can result in long-term biomechanical and anatomical alterations [13,14]. We hypothesized that the biomechanical alterations from the patient's previous acromioclavicular joint dislocation might have contributed to the development of her glenohumeral joint OA and subsequent osteophyte formation, culminating in her current symptoms. While the connection between past trauma and current symptoms remains a hypothesis in this case, the patient's improvement following the removal of bone fragments suggests that the impact of the glenohumeral joint OA was minimal. Since redness observed on arthroscopy is often associated with pain symptoms, we hypothesized that this was the cause of the patient's pain. This finding has been confirmed by previous studies and our own experience [15].

## Conclusions

We presented a case demonstrating a potential link between a traumatic injury occurring more than two decades prior and present-day symptoms. This case reinforces the importance of considering historical trauma in patients with atypical shoulder conditions. It serves as a reminder that shoulder disorders can present in unexpected ways, necessitating thorough clinical evaluation.
